# Does prediction error drive one-shot declarative learning?

**DOI:** 10.1016/j.jml.2016.11.001

**Published:** 2017-06

**Authors:** Andrea Greve, Elisa Cooper, Alexander Kaula, Michael C. Anderson, Richard Henson

**Affiliations:** MRC Cognition & Brain Sciences Unit, Cambridge, England, United Kingdom

**Keywords:** Prediction error, Associative memory, Encoding, One-shot learning

## Abstract

The role of prediction error (PE) in driving learning is well-established in fields such as classical and instrumental conditioning, reward learning and procedural memory; however, its role in human one-shot declarative encoding is less clear. According to one recent hypothesis, PE reflects the divergence between two probability distributions: one reflecting the prior probability (from previous experiences) and the other reflecting the sensory evidence (from the current experience). Assuming unimodal probability distributions, PE can be manipulated in three ways: (1) the distance between the mode of the prior and evidence, (2) the precision of the prior, and (3) the precision of the evidence. We tested these three manipulations across five experiments, in terms of peoples’ ability to encode a single presentation of a scene-item pairing as a function of previous exposures to that scene and/or item. Memory was probed by presenting the scene together with three choices for the previously paired item, in which the two foil items were from other pairings within the same condition as the target item. In Experiment 1, we manipulated the evidence to be either consistent or inconsistent with prior expectations, predicting PE to be larger, and hence memory better, when the new pairing was inconsistent. In Experiments 2a–c, we manipulated the precision of the priors, predicting better memory for a new pairing when the (inconsistent) priors were more precise. In Experiment 3, we manipulated both visual noise and prior exposure for unfamiliar faces, before pairing them with scenes, predicting better memory when the sensory evidence was more precise. In all experiments, the PE hypotheses were supported. We discuss alternative explanations of individual experiments, and conclude the Predictive Interactive Multiple Memory Signals (PIMMS) framework provides the most parsimonious account of the full pattern of results.

## Highlights

• Role of prediction errors (PE) in human one-shot declarative learning.
• PE is manipulated via previous experiences (priors) and sensory inputs (evidence).
• PE leads to superior memory across 5 different experiments.
• Support for Predictive Interactive Multiple Memory Signals (PIMMS).

## Introduction

Animals constantly extract regularities from past experiences to enable predictions about future events. Given that their environment is continuously changing, these predictions likewise need to adapt to novel information that may conflict with previously acquired expectations. The degree of conflict between predictions and new information is called prediction error (PE). PE plays a key role in many domains, such as reward learning, motivational control and decision making (Mackintosh, 1975, Pearce and Hall, 1980, Rescorla and Wagner, 1972, Schultz et al., 1997, Schultz and Dickinson, 2000, Sutton and Barto, 1998). Formal associative learning theories, for instance, state that learning is proportional to PE, where PE is the difference between expected and actual reward (Beesley and Shanks, 2012, Rescorla and Wagner, 1972). The recently proposed ‘Predictive Interactive Multiple Memory Signals’ (PIMMS) framework (Henson & Gagnepain, 2010) suggests that PE plays a general role throughout the human brain, in the service of both perception and multiple types of memory, from conditioning to perceptual priming and even declarative (e.g., episodic) memory.

Predictions are central to most computational models of learning. PE is the basis of the “delta” learning rule used in many connectionist models of human memory (Kinder & Shanks, 2001), though some have argued that such models cannot capture the one-shot learning that is characteristic of human declarative memory (Reber, 2002; although see Kinder & Shanks, 2003). More recent computational work suggests that the precision of predictions is an important determinant of the learning rate (e.g., Yu & Dayan, 2005), with the certainty of predictions possibly triggering a switch between gradual and rapid (e.g., one-shot) learning systems (Lee, O’Doherty, & Shimojo, 2015). Although PE is often taken for granted as a driver of learning, direct behavioural evidence for its role in one-shot learning of unique stimulus-stimulus associations, however, is scarce.

The most relevant work is by Le Pelley and colleagues (Griffiths and Mitchell, 2008, Le Pelley, 2010, Le Pelley et al., 2007, Le Pelley and McLaren, 2003), in particular their studies examining the impact of trained predictive and non-predictive cues on learning (Le Pelley, 2010). Across several experiments, these authors demonstrated more rapid discrimination learning when cues were previously established to be predictive. However, the content of the predictions was unrelated to the content of the information subsequently learned, leaving open the question of the role PE plays in learning. Moreover, most of these studies were based on animal learning paradigms, which differ in several ways from the paired-associate tradition often used to measure human declarative memory. Firstly, the animal learning paradigms normally involve multiple learning trials, rather than the single-trial learning. Secondly, these paradigms generally pair each stimulus with one of two outcomes (e.g., A+, B+, C−, D−, where + and − signify presence or absence of reward), rather than pairing two unique stimuli, as here (e.g., A−B, C−D, etc.). Perhaps most importantly, the animal paradigms tend to involve multiple stimuli (cues) associated with an outcome (e.g., AB+, AC−). With multiple cues, factors like selective attention become more important, in that participants can devote relatively more attention to those cues that are more predictive (Le Pelley, 2010; see also Kruschke, Kappenman, & Hetrick, 2005).

Other related work has investigated the effect of temporal context predictions on human memory, using repeated sequences or sub-sequences. These findings include better encoding into short-term memory for items that are less well predicted (Farrell & Lewandowsky, 2002) and better memory for items whose temporal context is repeated, even if the items themselves are not (Smith, Hasinski, & Sederberg, 2013), provided the prediction is strong (else the memory can actually be weakened, Kim, Lewis-Peacock, Norman, & Turk-Browne, 2014). However, none of this work has systematically tested long-term memory for multiple, unique stimulus-stimulus associations, nor manipulated PE by independently varying the precision and the accuracy of predictions.

PIMMS is a general framework for understanding how prior knowledge influences the perception and acquisition of new information. The brain is assumed to contain hierarchical representations of the world, where representations active at one level of the hierarchy predict the activity of representations in lower levels. The difference between those predictions and the sensory evidence from lower levels comprises the PE, and this PE is assumed to drive synaptic change (learning) between levels, so as to improve predictions and ultimately minimise PE in future (Friston, 2005, Mumford, 1992). PIMMS does not specify the precise mechanism by which PE drives learning, e.g., whether that be “local” PE in a delta-learning rule that updates individual weights, “global” PE that modulates overall learning rate across all weights, or even whether PE only triggers increased attention, and it is attention that actually mediates memory encoding. What PIMMS offers is a Bayesian framework for considering how PE might vary in the world, and therefore be manipulated experimentally in the laboratory. As an example, PIMMS assumes that walking into a familiar room activates a representation of that room, which in turn predicts what objects are expected inside the room. Objects that are present and predicted do not produce a PE, and no learning results; an unexpected (but familiar) object, however, produces a PE, which causes learning to update the predictions so that the object becomes more expected in that room in future. Walking into a completely novel room, on the other hand, does not generate predictions, so a novel object in a novel room will not be encoded because there is no PE (despite the maximal novelty of the situation). A familiar object in a novel room, on the other hand, does produce a PE and its association with that room will be learned faster than a novel object.

**Fig. 1:**
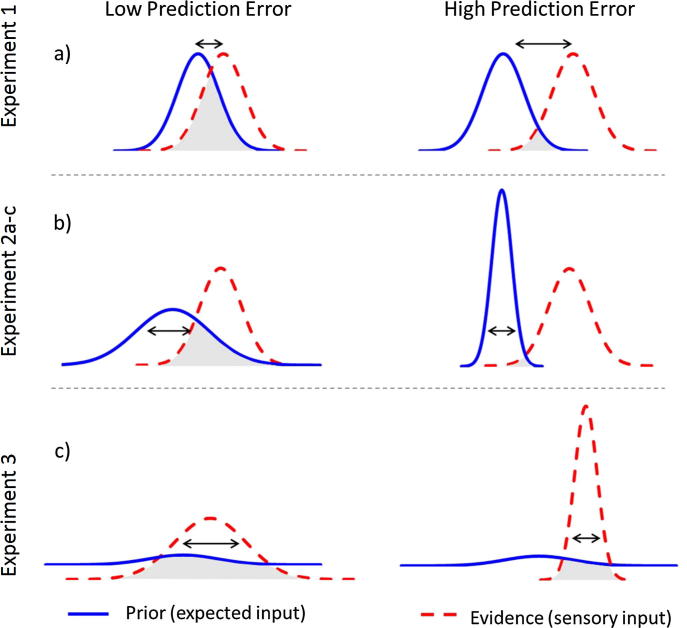
Changes in Prediction Error as a result of modulating the difference between the mean of the prior and evidence (Experiment 1), modulating the precision of prior expectations (Experiment 2a–c) or modulating the precision of the sensory evidence (Experiment 3). The curves represent probability distributions over a hypothetical dimension along which stimuli vary in similarity (*x*-axis). The blue, solid line represents the prior probability of one stimulus (e.g., word or face) given a cue (e.g., scene), while the red, dotted line represents the sensory evidence (likelihood) for that stimulus. Prediction Error is the divergence (lack of overlap) between these two distributions (i.e., greater when shaded area of overlap in prior and evidence is smaller). (For interpretation of the references to colour in this figure legend, the reader is referred to the web version of this article.)

In the present study, we set out to provide evidence for this core assumption of PIMMS that PE drives one-shot learning within a more typical human associative memory (paired associate) laboratory paradigm. We conducted five behavioural experiments that measured memory for a single pairing of two visual stimuli, as a function of the prior history of those stimuli. According to PIMMS, PE can be defined as the divergence between an expected outcome (prior) and an observed outcome (evidence); also called the “Bayesian Surprise” (Friston, 2010). Assuming unimodal probability distributions, Fig. 1 illustrates three ways in which this PE can be manipulated. Firstly, one can vary the accuracy of the prediction, i.e., the difference between the modes of the prior and evidence distributions, with a larger difference producing greater PE (Fig.1a). This is akin to a room predicting a familiar object that is different from the one encountered there. This is the approach we took in Experiment 1, by establishing predictions (priors) for the valence of a word given a category of scenes in a Training Phase, and then testing associative memory for new scene-word pairings presented in a second “Study” phase, where the new word (evidence) was either consistent (low PE) or inconsistent (high PE) with the valence predicted by the trained scene category. Importantly, we tested memory with three-alternative forced choice (3AFC), in which all the response options came from the same Study phase, in order to prevent proactive interference from the Training phase.

A second way to manipulate PE is to vary the precision of the prediction (i.e., sharpness of the prior distribution; Fig.1b). This is akin to varying the familiarity of the room that contains a familiar (but unexpected) object and is the approach we took in Experiments 2a–c. More specifically, we paired a scene with (i) a single face multiple times, (ii) a single face only once, or (iii) multiple different faces. The ability to associate these scenes with completely new faces was subsequently tested using 3AFC. Better memory performance was expected when the scene had previously established a more precise prediction for a specific face.

The final way in which we manipulated PE in Experiment 3 was by varying the precision of the evidence (Fig.1c). We did this in two ways: (1) by adding visual noise to unfamiliar faces (assumed to decrease the precision of the evidence) and (2) priming a subset of them in advance (assumed to increase the precision of the evidence). When pairing these with an arbitrary scene (with no prior predictions), we expected better scene-face memory (i.e., 3AFC performance) for the primed and noise-free faces. This example is akin to varying the familiarity of an object appearing in a novel room.

## Experiment 1

Experiment 1 varied the accuracy of the priors, in terms of their difference from the evidence. First, participants were trained to associate particular categories of everyday scenes with categories of words describing a positive or negative emotional valence. In a later Study phase, novel scenes from the same categories were paired with novel words whose valence was either the same (consistent, or “Low PE” condition) or opposite (inconsistent, or “High PE” condition) to that originally associated with the scene category in the Training phase. Trials of both conditions were intermixed, and interspersed with other consistent trials, ensuring that predictions were generally maintained. To confirm that expectations were established, participants provided explicit predictions regarding the valence of the word expected to be paired with a scene. In the Test phase, a 3AFC test was used to assess how well the new scene-word pairings were encoded during the critical Study Phase, by providing a scene and a choice of three words. Importantly, the 3AFC foil words were (1) from the same condition as the target item, i.e., either all from the Low PE condition or all from the High PE condition, (2) presented in other trials of the Study phase, and (3) of the same valence. These constraints meant that above-chance 3AFC performance required memory for the specific Study phase pairing, i.e., could not be solved by general knowledge of the word category (valence) associated with the scene in training, nor by familiarity with the individual words. We predicted that 3AFC performance would be better for inconsistent than consistent pairings, i.e., better in High PE than Low PE conditions.

### Methods

#### Participants

Twenty Cambridge community members were recruited from the volunteer panel of the MRC Cognition and Brain Science Unit (12 females, mean age 26), all of whom had reported normal or corrected-to-normal visual acuity, provided informed consent and received monetary compensation for participation, as approved by a local ethics committee (Cambridge Psychological Research Ethics Committee reference 2005.08).

#### Procedure

**Fig. 2:**
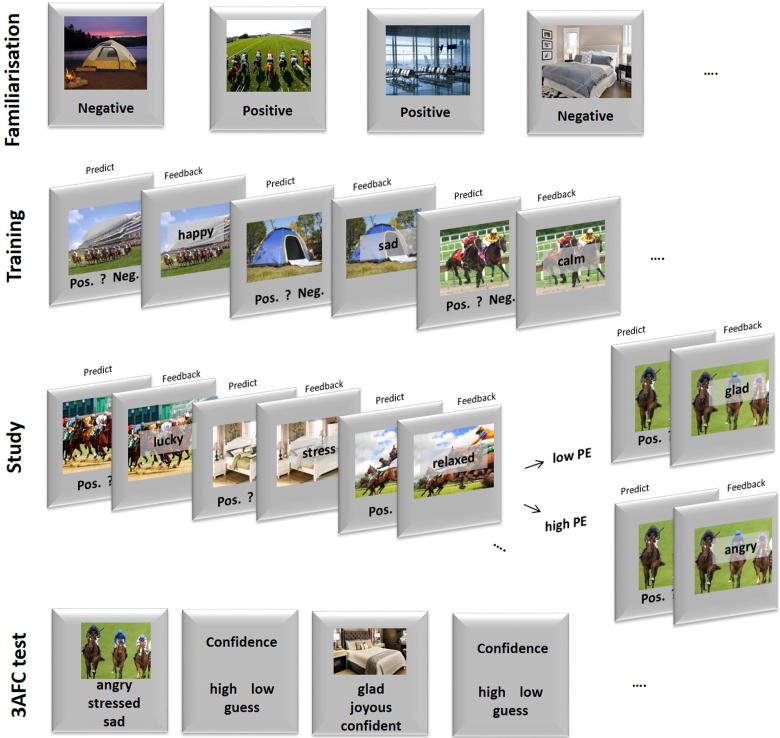
Design of Experiment 1. An initial Familiarisation phase informed participants about which scene category was associated with positive or negative valence through passive ‘Watch’ and active ‘Predict’ blocks. In the subsequent Training phase, new scenes from the same categories were paired with specific words (adjectives) that possessed the same valence as that given in the Familiarisation phase. In the critical Study phase, new scenes were again paired with new words of the same valence as Training, but on the third time a scene category was presented, the valence of the paired word was either switched from that experienced during Training/Familiarisation (inconsistent: High PE condition) on one half of trials, or remained (consistent: Low PE condition) on the other half. Because these “critical” Study trials were intermixed with “filler” trials of first and second presentations of a scene category in the Study phase (for which the valence of the paired word was always consistent with Training/Familiarisation), the overall proportion of inconsistent trials was only 1/6th of trials in the Study phase. Memory for the pairings in the critical trials of the Study phase was assessed in the final Test Phase, which used 3AFC in which the target word was offered together with two foil words that were of the same valence and encountered in other trials of the same condition during the critical Study Phase.

The procedure consisted of four phases, as illustrated in Fig. 2, with each phase detailed below.

#### Familiarisation phase

The Familiarisation phase served to establish participants’ scene-valence expectations. 48 pictures each from unique categories were presented in 4 cycles of 12 new trials, with each cycle containing a ‘watch’ block and at least one ‘predict’ block. The ‘watch’ block, presented a scene with either the word ‘positive’ or the word ‘negative’ for 2000 ms with the instruction to memorise the pairings. The ‘predict’ blocks presented the same 12 scenes in random order, and participants pressed a key to indicate whether each was associated with ‘positive’ or ‘negative’. Following the response, a blank screen appeared for 100 ms and the correct valence was superimposed on the scene for 1000 ms. A final predict block was run in which all 48 scenes were presented.

#### Training phase

The Training phase transferred the general positive/negative valence learned in the Familiarisation phase to novel images of scenes from the same categories. The procedure resembled the ‘predict’ blocks from the Familiarisation phase. The response options remained ‘positive’ or ‘negative’, but feedback was now given with specific adjectives of positive or negative valence matching the valence presented during familiarisation (e.g., ‘sad’, ‘happy’; see Materials).

#### Study phase

The Study phase presented a continuous stream of trials constructed from a series of mini-blocks intermixing two training and one ‘critical’ Study trial for all 48 categories. The procedure remained identical to the ‘predict’ block in the two previous phases. In ‘critical’ Study trials previously trained valence-scene associations were either switched (inconsistent: High PE condition) or maintained (consistent: Low PE condition). Two-thirds of all trials were training trials that maintained their previous valence association. Since Low PE critical study trials also conformed to expectations, violations only occurred for High PE critical study trials (1/6 of all trials). Because the majority (5/6) of trials, therefore, maintained their trained association, participants would be encouraged to make use of their acquired knowledge and predictions. The number of trials intervening between High PE trials ranged from 0 to 10, with a mean of 5. Training trials for certain scenes were intermixed with critical Study trials for other scenes so that the latter were not all presented at the end of a block. Critical trials were always paired with unique adjectives whereas training trials were paired with one of eight specific adjectives (see Materials).

#### Test phase

The Test Phase assessed associative memory for the scene-word pairings of critical Study trials using a 3AFC test. A scene was presented at the centre of the screen with three words below it. One of the words was the correct choice (target); the other two words (foils) had the same valence as the target but had been paired with different scenes in the critical Study phase. All three alternative options were selected from the same condition (i.e., either the High PE or Low PE condition), and appeared three times during the Test Phase: once as the target, and twice as a foil, with the order of these occurrences randomised. The three choices remained on the screen until participants selected a word via a button press. Finally, participants indicated how confident they were of their choice via a button press (high, low, and guess).

#### Materials

Stimuli consisted of colour photographs and words. The photographs were selected to display 48 categories of indoor and outdoor scenes with 6 distinct images per category, i.e., 288 images in total. Categories were randomly paired with a valence (positive or negative) in the Familiarisation phase. Word stimuli were constructed from 58 adjectives of positive or negative valence. Scenes were divided into 6 sets; each set contained 48 unique scenes, one per category. During Training these scenes were paired with a specific word of a valence consistent with the Familiarisation phase. For critical Study trials, one-half of these scenes were paired with a word violating this valence. Scenes and words were counterbalanced (for precise details of scene-word pairings, see Appendix A).

#### Analysis

In line with the PE hypothesis, the central prediction was that 3AFC performance would be better when prior predictions are violated, i.e., more items remembered in the High than Low PE condition. This hypothesis was therefore tested with a paired *t*-test with two-tailed alpha = .05. Performance was measured by proportion of correct trials in the Test phase (chance = .33). To examine declarative memory, analyses focussed on high and low confidence responses but omitted self-reported guesses. There was no significant difference in the number of guesses between conditions, *t*(19) = 1.67, *p* = .11, ranging from 0 to 12 (Mean = 5.90) in the Low PE condition and from 0 to 13 (Mean = 6.85) in the High PE condition. Separating performance by high vs. low confidence did not add any new information; therefore performance is reported collapsed across confidence.

### Results

#### Performance during training and study

Mean prediction accuracy was high during both Familiarisation Predict blocks (*M* = .88) and Training blocks (*M* = .86), and did not differ significantly between High and Low PE conditions during the Familiarisation phase, *t*(19) = 0.60, *p* = .55, nor during the Training phase, *t*(19) = 0.89, *p* = .38. At this stage, no difference was expected because the conditions were identical. For the critical Study trials, predictions were highly accurate in the Low PE condition (*M* = .91) where valence remained the same for critical trials, but not in the High PE condition (*M* = .03) because the contingency was reversed in the High PE condition.

#### Memory test performance

Performance on the associative memory 3AFC test is shown in Table 1. Mean performance was significantly better in the High PE condition than Low PE condition, *t*(19) = 2.54, *p* = .01, with a mean difference of .06 (95% confidence interval from .01 to .11), corresponding to a medium Cohen effect size of *d* = .57.

### Discussion

Experiment 1 manipulated PE by training participants to expect a word of a particular valence to follow a scene of a given category. The critical Study trials contained new scene-word associations either consistent (Low PE) or inconsistent (High PE) in valence with the trained expectations. In keeping with our hypothesis, memory for the new critical scene-word pairing was better in the High than Low PE condition. This is consistent with the PIMMS framework proposing that PEs not only drive incremental associative learning, but also one-shot declarative memory. We discuss other interpretations of this finding in the General Discussion.

First, we report further experiments that manipulated PE in a different way. In Experiments 2a–c, rather than manipulating the degree to which the sensory evidence matches the priors, we fixed the difference between the sensory evidence and the prior, and manipulated instead the precision of the prior (see Fig.1b), reasoning that more precise priors will lead to greater PE when they are violated.

## Experiment 2a

In Experiments 2a–c, we manipulated the prior expectation by training participants to associate a scene with one or more unfamiliar faces. Experiment 2a tested three conditions: High PE, Baseline, and Low PE. For Training trials in the High PE condition, a scene was repeatedly paired with the same face during Training (forming a precise prior); in the Baseline condition, a scene-face pair was only presented once (forming a less precise prior); while in the Low PE condition, a scene was paired with different faces (forming the least precise prior).

The PE itself occurred in later Study trials when the scenes were paired with completely new and unpredicted faces in all conditions. Associative memory for these Study trials was tested using 3AFC, as in Experiment 1, with response options restricted to Study trials only, in order to minimise proactive interference from Training trials. We expected best 3AFC performance in the High PE condition and worst performance in the Low PE condition.

For both Training and Study trials, participants made a speeded male/female decision about that face. Reaction times (RTs) for this decision provided an indirect measure of participants’ predictions, i.e., speeding up during Training trials showing the same face, and slowing down between the final Training trial and the Study trial, when predictions were violated. We therefore tested whether this RT difference correlated with subsequent memory. Trials of all conditions were intermixed into a continuous sequence of scene-face pairs, such that participants were not informed of any difference between Training and Study trials.

### Methods

#### Participants

Twenty-eight participants were recruited using the same procedure as described in Experiment 1. Four participants were excluded as outliers because their memory performance pooled across all three conditions deviated more than two standard deviations from the group mean. Final analysis included a fully counterbalanced dataset of twenty-four participants (17 female, mean age 25).

#### Material & procedure

**Fig. 3:**
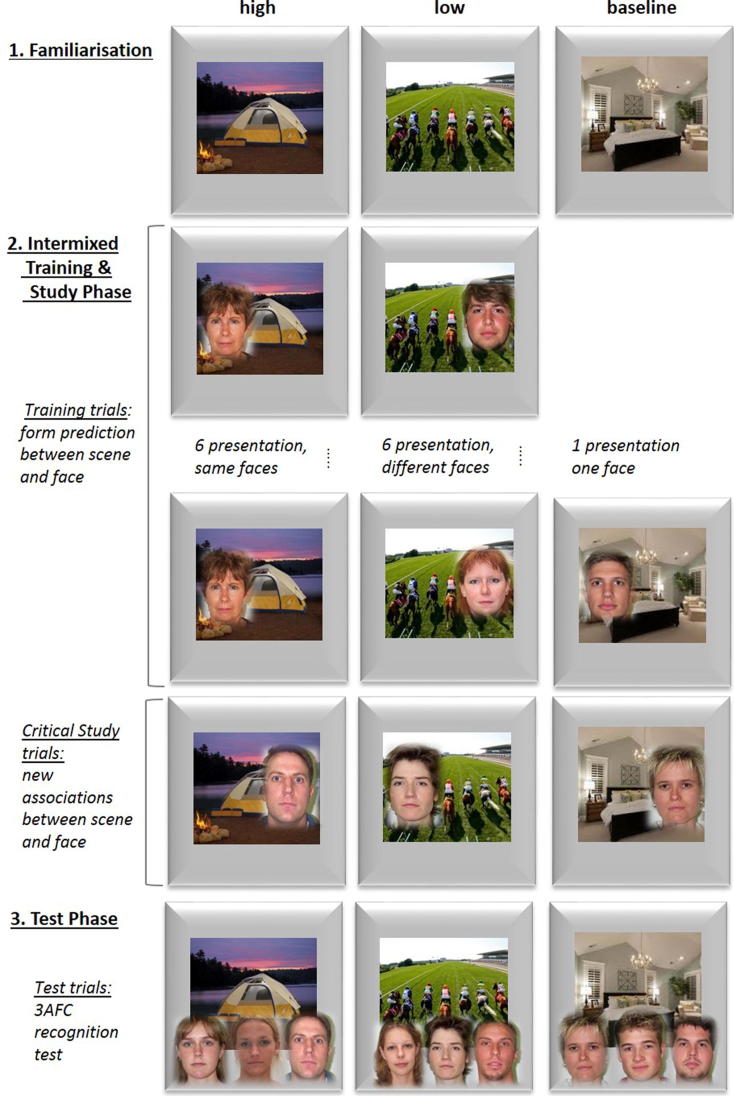
Design of Experiment 2a showing a single run. At the start of each run, all scenes were presented twice to familiarise participants with the images. Participants kept count of indoor/outdoor scenes. The Training trials then established either strong priors, by repeatedly presenting the same scene with the same face (High PE condition), weaker priors, by presenting a scene-face pair only once (Baseline condition) or least strong priors, by presenting the same scene with different faces (Low PE condition). Critical Study trials (intermixed with Training trials) used novel faces, evoking the different degrees of prediction error. Participants made speeded male/female decision on the faces. In the Test phase, associative memory for faces paired in the critical Study trials was assessed, using 3AFC in which the foils came from other critical Study trials.

Stimuli consisted of 108 photographs of indoor and outdoor scenes and 396 photographs of male and female faces. For Training trials, scenes were repeated 6 times for High and Low PE conditions but were only presented once in the Baseline condition. Scene stimuli were divided into 3 sets of 36 images and randomly allocated to the 3 conditions (one set for High PE, Low PE, and Baseline). Face stimuli were randomly divided into 8 training sets of 36 images and allocated to the 3 conditions: one to High PE (one face was repeated six times with the same scene), six to Low PE (six different faces were presented once each with the same scene) and one for Baseline (one face was shown with one scene only once). Three additional sets of 36 faces were created, one per condition, which served as critical Study trials to be paired once with all previously trained scenes. Previously trained scenes were now paired with new faces at Study to violate the training predictions and evoke different degrees of PE across conditions (for counterbalancing of sets see Appendix B). The experiment was divided into 6 runs, with each run using 18 unique scenes (6 scenes per condition). The procedure for one run is illustrated in Fig. 3 and described below. Participants performed an additional practice block of 6 trials with extra stimuli in order to become familiar with the procedure.

#### Familiarisation phase

In the initial Familiarisation phase, each scene appeared twice in a random order, while participants kept a covert running count of the number of indoor scenes, which had to be reported at the end of the phase to ensure participants were watching the stimuli. Each scene was presented for 1000 ms in the centre of the screen, separated by a 250 ms blank screen. The purpose of this phase was to familiarise participants with each scene, in order to minimise differences between the Baseline and High/Low PE conditions in terms of scene familiarity.

#### Training and study phase

For Training trials, six scenes were paired six times with the same face (High PE condition), six scenes were paired six times with a different male or female face each time (Low PE condition), and six scenes were presented with a face only once (Baseline condition). These conditions were intermixed within a run. Each Train/Study trial started with a blank screen for 500 ms, followed by a scene in the centre of the screen for 1000 ms (allowing participants time to generate expectancy for the subsequent face). A face was then superimposed on the screen for another 1000 ms. The face occurred on the top or bottom of the screen (an equal number of times). This top/bottom location was consistent across the six repetitions of the same face during High PE Training trials, but alternated with each new face during Low PE Training trials. Half of the faces were male and the other half female, which alternated across Low PE trials. Participants were instructed to make speeded gender judgement using the assigned response keys that were counterbalanced across participants. Participants were aware that their memory would be tested later, but were informed that this was only a secondary goal of the study. Their primary focus was to acquire gender judgements as fast and as accurate as possible.

Mini-runs of 2–3 scenes from each condition were interspersed, so that Study trials for a particular scene always occurred after its Training trials, but Study trials for some scenes could occur before Training trials for other scenes. Participants were not informed of the difference between Training and Study trials. An average lag of 11 intervening trials separated the final Training trial and the critical Study trial for a given scene. The gender and/or location of the face in the Study trial was switched relative to that in the final Training trial for half of the Study trials, but remained the same for the other half.

#### Test phase

After the Training/Study phase, participants performed an odd/even distractor task for 40 s, to reduce any contribution of short-term memory. A random number between 0 and 99 occurred on the screen, and participants judged whether the number is odd or even, indicating their choice via a button press.

Memory for face-scene pairs from critical Study trials was assessed using a 3AFC test similar to Experiment 1. Each scene was presented with three faces below it – the target and two foils – and the participants’ choice was followed by an indication of high or low confidence, using one of two keys. Participants then were prompted to recall the location (i.e., top/bottom) of the face. This “Context Memory” decision was made using a button-press in a four-way judgment of “think top, guess top, guess bottom or think bottom”.

#### Analysis

The main hypothesis was for associative memory 3AFC performance to increase as the precision of predictions, and hence size of PE, increased. Two pairwise *t*-tests were used: (1) Low PE vs. Baseline and (2) Baseline vs. High PE. Separating performance by high vs. low confidence did not add any new information, nor did analysis of the context memory decisions; therefore performance was collapsed across these factors.

### Results

#### Associative memory

Performance on the associative memory 3AFC test is shown in Table 1. As expected, mean performance was best in the High PE condition, and worst in the Low PE condition. Pairwise tests confirmed significantly better performance in the Baseline than Low PE condition, *t*(23) = 2.93, *p* = .01, *d* = .39 (mean difference: .04; 95% confidence interval from .005 to .08), though any difference between High PE and Baseline conditions did not reach significance, *t*(23) = .68, *p* = .50, *d* = .14 (mean difference: .02, 95% confidence interval from −.02 to .06).

#### RTs during training and study

**Fig. 4:**
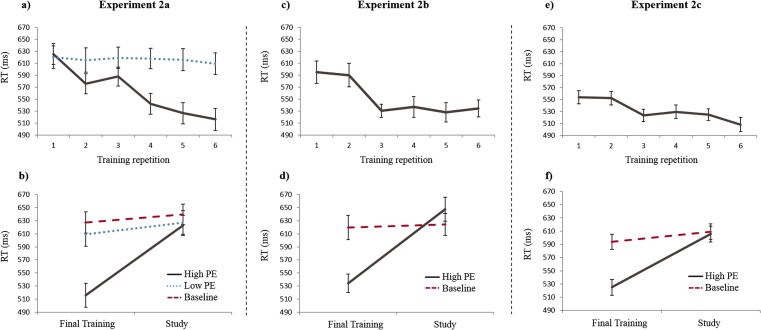
Reaction times (RTs) for (a + b) Experiment 2a, (c + d) Experiment 2b and (e + f) Experiment 2c. The top panels (a, c, e) show RTs across multiple Training repetitions for High PE (brown, solid) and Low PE (blue, dotted) conditions. The bottom panels (b, d, f) show RTs for final Training and critical Study trials, which were intermixed (i.e., the *x*-axis implies temporal order only for single scene, but not across different scenes). Baseline condition (red, dashed). Error bars are standard errors. Final Training in the bottom panel is also Training trial 6 in top panel. (For interpretation of the references to colour in this figure legend, the reader is referred to the web version of this article.)

Reaction times as a function of Training trials (1–6) and critical Study trial are shown in Fig.4a. There was a significant linear decrease in RTs across the six Training trials in the High PE condition, *t*(23) = 11.5, *p* < .001, but not in the Low PE condition, *t*(23) = .96, *p* = .34, as would be expected since High PE scene-face pairs remained the same, whereas Low PE faces were new for each trial. There was a significant slowing in RTs from the final (sixth) Training trial to the Study trial in the High PE condition, *t*(23) = 13.1, *p* < .001, and in the Low PE condition, *t*(23) = 3.68, *p* = .001, as well as from the first (and only) Training trial to the Study trial in the Baseline condition, *t*(23) = 2.73, *p* = .01 (Fig.4b). The degree of this slowing was greater in the High PE condition than either of the other two conditions, *t*(23) > 9.15, *p* < .001. Finally, RTs for the critical Study trial did not differ between High and Low PE conditions, *t*(23) = .67, *p* = .51, though were slower in the Baseline condition than in both of the other conditions, *t*(23) > 2.46, *p* < .01.

#### Correlation between RT slow-down and associative memory

**Fig. 5:**
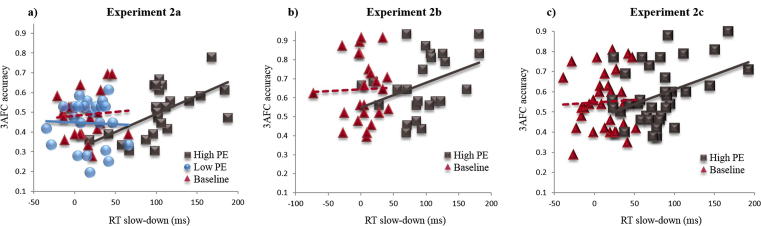
Scatter plot of associative memory performance against slow-down in RTs between final Training and critical Study trial for (a) Experiment 2a, (b) Experiment 2b and (c) Experiment 2c. Regression lines are superimposed for the baseline condition (dashed red line), High PE condition (solid brown line) and Low PE condition (solid blue line). (For interpretation of the references to colour in this figure legend, the reader is referred to the web version of this article.)

If the slow-down in RTs between the final Training trial and the critical Study trial indexes the amount of prediction error, then we would expect this to correlate positively with the amount of learning, i.e., associative memory performance. This RT slow-down (Final Training RT minus Critical Study RT) is plotted against associative memory performance for each participant and condition in Fig.5a. The correlation was significantly positive in the High PE condition, *r*(23) = .57, *p* = .004, but did not reach significance in either the Baseline, *r*(23) = .08, *p* = .72, or Low PE, *r*(23) = −.04, *p* = .87, conditions. Note that associative memory did not correlate with raw RTs of critical Study trials in any of the three conditions, including the High PE condition, *r*(23) < .08, *p* > .71, and the correlation between associative memory and the difference between Training and critical Study RTs in the High PE condition remained significant after partialling out critical Study RTs, *r*(21) = .57, *p* = .004. These findings demonstrate that it is the slow-down between Training and critical Study trials (presumably reflecting prediction error) that determines memory, rather than simply the amount of time spent processing the novel faces in the critical Study trials.

### Discussion

Experiment 2a examined the effect of the precision of the priors, i.e., degree to which a given scene predicted a face, on encoding of a new pairing of that scene with a novel face. As hypothesized, memory for the new scene-face pairing was better when the erroneous prior was more precise, consistent with a larger prediction error. This memory advantage was reflected by significantly better 3AFC associative memory performance in the High PE and Baseline conditions than the Low PE condition. Moreover, a more direct measure of prediction error – namely the slow-down in RTs for face gender judgements in critical Study trials relative to final Training trials – correlated positively with associative memory within the High PE condition, and this correlation was not driven simply by RTs for the critical Study Trials.

There was a speed-up in RTs across the six Training trials in the High PE but not Low PE condition. While this is consistent with participants making predictions about the face that follows a given scene in the High PE condition, it is also possible that this speed-up simply reflects more fluent processing of the (repeated) face itself, regardless of the preceding scene-prediction. In other words, this RT speed-up could reflect repetition priming of faces in the High PE condition, which could not occur in the Baseline or Low PE conditions. This repetition priming could reflect tuning of a perceptual representation of the face (resulting in a more precise evidence distribution, as assumed by PIMMS in Fig.1C and Experiment 3). Alternatively, the RT speed-up could reflect retrieval of stimulus-response (S-R) bindings, i.e., stimulus-specific cueing of the previous male/female response, without any change in the stimulus representation itself (Henson, Eckstein, Waszak, Frings, & Horner, 2014). Any of these accounts would also predict the slow-down in RTs for the novel face in the critical Study trial of the High PE condition (though the fact that the gender of the faces used in the final Training trial and Study trial switched on 50% of trials, i.e., gender was only compatible half of the time, may have attenuated any influence of S-R bindings). Importantly, however, only the scene-prediction (PE) account naturally explains why the size of this slow-down between Training and Study correlates with subsequent memory for the novel scene-face pairing in the High PE condition.

According to PIMMS, there is no reason why this correlation between RT slow-down and subsequent memory should not also occur in the Baseline or even Low PE condition if one assumes that there is variation across participants in how well a scene can be associated with a face (following one Training trial). One reason why this correlation did not reach significance in the Baseline and Low PE conditions may be simply the limited range of this variation, relative to the larger range induced by reinforcing predictions across multiple Training trials in the High PE condition. Another reason may be because the RT slow-down between Training and critical Study in Baseline and Low PE conditions also includes other components that swamp individual differences in predictions. For example, it is possible that participants learned, to varying degrees through the course of the experiment, that changes occur after the first or sixth presentation of a scene. This may have caused some participants to slow down in anticipation of that change, in all conditions, and this general slow-down may not necessarily improve memory.

Finally, though the pairwise test of 3AFC memory between Baseline vs. Low PE conditions was significant, the High PE vs. Baseline comparison did not reach significance. It is important to establish whether memory is better in the High PE than Baseline condition because worse performance of the Low PE condition could potentially be explained by proactive interference. More precisely, the 3AFC test only offered faces from Study trials, so that none of the potentially interfering faces that were paired with the same scene in Training trials were a response option. Although this controls for overt proactive interference, it is possible that interfering faces from Training trials still “come to mind” and impair target selection in the 3AFC test trials (cf. the “fan effect”, Radvansky, 1999, Schneider and Anderson, 2012, Sohn et al., 2004). Indeed, such covert retrieval of trained faces could have occurred during Study trials too, impeding learning of the new face association. Such interference could explain the worse performance in the Low PE condition relative to Baseline condition, in that up to six competing faces could intrude in the Low PE condition, but only one could intrude in the Baseline condition. However, any such covert interference from the trained faces would also be greater for the High PE condition (when it is paired six times) than Baseline condition (when it is paired only once), predicting better, not worse, performance in the Baseline than High PE condition. Hence Experiment 2b re-tested the comparison of Baseline and High PE conditions.

## Experiment 2b

The purpose of Experiment 2b was to test whether the small numerical advantage in associative memory for High PE relative to Baseline conditions in Experiment 2a would be significant when statistical power was increased. We therefore omitted the Low PE condition, in order to increase the number of trials in the remaining two conditions. Without the intermixed trials of the Low PE condition, in which the face paired with a given scene changed frequently, we were concerned that participants might be able to predict when the face would change in the High PE condition (i.e., learn that once a scene-face pairing had been repeated once, it would be presented six times in total before changing in the critical Study trial). To increase the uncertainty about when a face pairing would change, we split the High PE condition into two sub-conditions in which the pairing changed after either 4 or 6 Training trials (henceforth, the High PE4 and High PE6 conditions).

A further change from Experiment 2a was that the gender of the face always switched between Training and Study trials. This should reduce the RT variability induced by compatible versus incompatible S-R bindings between Training and Study trials that may have occurred in Experiment 2a.

### Methods

#### Participants

Twenty-seven participants were recruited, using the same procedure as in Experiment 1. Three participants were excluded as outlier because their memory performance pooled across all three conditions deviated more than two standard deviations from the group mean. Final analysis included a fully counterbalanced dataset of twenty-four participants (13 females, mean age 23).

#### Material & procedure

Stimuli consisted of 96 photographs of indoor and outdoor scenes and 192 photographs of male and female faces, a subset of those used in Experiment 2a. Stimuli were constructed similar to Experiment 2a with the exception of omitting the Low PE condition. The current experiment, therefore, contained 2 conditions: a Baseline condition and a High PE condition. The number of Training presentations in the High PE condition was either 4 or 6 (for details of scene-word pairings see Appendix C).

The experiment was divided into 6 runs, each presenting 16 unique scenes for Training, Study and Test trials (8 scenes per condition). Participants performed an additional practice block of 6 trials in order to become familiar with the procedure.

The procedure for one run was similar to Experiment 2a, in which all conditions were intermixed. At the start, all scenes in that run were presented twice in random order to familiarise the participants with the images. The Training trials for the High PE condition consisted of 24 unique scenes-face pairs, presented 4 times; and another 24 unique scenes-face pairs, presented 6 times. For the Baseline condition, the Training trials consisted of 48 unique scenes-faces pairs shown only once. Study trials were intermixed into the continuous stream of Training trials, with an average lag of 11 intervening trials between trials containing the same scene image. The location of the face on the scene always switched between final Training and critical Study trials.

Following the odd/even distractor task, a 3AFC test of associative memory was presented with an identical procedure and trial structures as in Experiment 2a, including confidence judgements. Again, separating performance by high vs. low confidence did not add any new information, and so performance was collapsed across it.

### Results

#### Associative memory

As planned, for analysis of associative memory, we collapsed across High PE4 and High PE6 conditions to equate their trial numbers with those in the Baseline condition and hence maximise power. The results are shown in Table 1. Overall performance was higher than in Experiment 2a, most likely because fewer new face-scene associations were presented during each “mini-run” of Training and Study trials. As predicted, memory was better for the High PE than Baseline condition, but statistical comparisons revealed only marginally significant differences *t*(23) = 2.00, *p* = .058, *d* = .41 (mean difference: .03; upper and lower 95% confidence interval: −.055 and .001).

#### RTs during training and study

Reaction times as a function of Training trials and the critical Study trial are shown in Fig.4c. There was a significant linear decrease in RTs across Training trials collapsing across High PE conditions, *t*(23) = 7.15, *p* < .001. Significant slowing in RTs from the final Training trial to the critical Study trial was present in the High PE condition, *t*(23) = 10.27, *p* < .001, but not the Baseline condition, *t*(23) = .91, *p* = .37. The degree of this slowing was significantly greater in High PE conditions relative to the Baseline condition, *t*(23) = 7.58, *p* < .001 and mean RTs for the critical Study trial did not differ significantly across conditions *t*(23) = 1.78, *p* = .088 (Fig.4d).

#### Correlation between RT slow-down and associative memory

The slow-down in RTs between the final Training trial and the critical Study trial is plotted against associative memory for each participant and condition in Fig.5b. A significant positive correlation was again found in the High PE condition, *r*(23) = .38, *p* = .04, but not in the Baseline condition, *r*(23) = .03, *p* = .89. Analogous to Experiment 2a, associative memory did not correlate with raw RTs of critical Study trials in either condition, *r*(23) > −.22, *p* = .29 and *r*(23) < .15, *p* = .47, but was significantly correlated with the slowdown in RTs between the final Training and critical Study trials in the High PE condition, even after partialling out critical Study RTs, *r*(21) = .57, *p* < .01, again demonstrating that memory does not depend simply on the amount of time spent processing the novel faces in the critical Study trials.

### Discussion

Though the difference between memory performance in the High PE and Baseline was in the predicted direction, this difference did not quite reach conventional two-tailed significance levels. We therefore postpone discussion until replicating the effect in the more highly-powered Experiment 2c.

## Experiment 2c

Experiment 2c was a nearly exact replication of Experiment 2b, except for the minor change that context memory was no longer assessed, which meant that faces could be presented in the middle of the screen at Study (rather than at top or bottom). Importantly, based on the High PE versus Baseline effect size in Experiment 2b, we powered the study to have a 75% probability of detecting a significant memory advantage by using 36 participants (the next number of participants after 24 that allowed full counterbalancing).

### Participants

Thirty-six participants (25 female, mean age 24) were recruited, using the same procedure as in Experiment 1.

### Material & procedure

These were the same as Experiment 2b.

### Results

#### Associative memory

As planned, we collapsed across High PE4 and High PE6 conditions (results are shown in Table 1). Overall memory performance was worse than in Experiment 2b, for reasons that are unclear and may simply relate to different participant samples. Nonetheless, the within-participant comparison of High PE versus Baseline conditions was in the same direction and of similar magnitude, and importantly now reached significance, *t*(35) = 2.37, *p* = .023, *d* = .22 (mean difference: .03; upper and lower 95% confidence interval: .06 and .005).

#### RTs during training and study

Reaction times as a function of Training trials and the critical Study trial are shown in Fig.4e and 4f. The results replicated those of Experiment 2b, with a significant linear decrease in RTs across Training trials, collapsing the High PE conditions, *t*(23) > 7.50, *p* < .001. There was a significant slowing in RTs from the final Training trial to the critical Study trial in the High PE conditions, *t*(23) = 9.44, *p* < .001, and also the Baseline condition, *t*(23) = 2.59, *p* = .014. The degree of this slowing was significantly greater in the High PE conditions than Baseline condition, *t*(35) > 5.71, *p* < .001 and mean RTs for the critical Study trial did not differ significantly between conditions, *t*(35) < 0.79, *p* > .43.

#### Correlation between RT slow-down and associative memory

The slow-down in RTs between the final Training trial and the critical Study trial is plotted against associative memory for each participant and condition in Fig.5c. Again, the results replicated those of Experiment 2b, with a significant positive correlation in the High PE condition, *r*(35) = .43, *p* < .01, but not in the Baseline condition, *r*(35) = .04, *p* = .83. Associative memory did not correlate with critical Study trial RTs in either condition, *r*(35) < .04, *p* = .81 and *r*(35) < .14, *p* = .42, but remained significantly correlated with the slow-down in RTs between final Training and critical Study trials in the High PE condition even after partialling out critical Study trial RTs, *r*(33) = .44, *p* < .01, again demonstrating that memory did not depend simply on the amount of time spent processing the novel faces in the critical Study trials.

### Discussion

By adding more trials and testing more participants to boost power, Experiment 2c confirmed that associative memory is better in the High PE condition than Baseline condition, supporting the trends in Experiment 2a and 2b. This is important because it challenges the alternative explanation of Experiment 2a’s results in terms of proactive interference, since the High PE condition should have suffered from more proactive interference than the Baseline condition, owing to the stronger association from the greater number of Training trials. Furthermore, both Experiments 2b and 2c replicated the slow-down in RTs between Training and critical Study trials in the High PE condition, and the correlation of this slow-down with subsequent associative memory. These results are consistent with the hypothesis that participants make predictions for the faces that follow each scene, which are subsequently violated in the critical Study trials. The degree of this violation (depending on the strength of the prediction) predicts how well the new scene-face pairing will be remembered later. We return to this and other interpretations in the General Discussion. First, we report a final approach to manipulating PE, this time by varying the precision of the evidence distribution, without changing the priors.

## Experiment 3

Experiments 3 again tested the one-shot encoding of novel scene-face pairs, but this time by manipulating the sensory evidence for the faces by (i) perceptual degradation and (ii) priming. In contrast to Experiment 2, scenes were presented only once at encoding, so participants were unable to predict the subsequent face (corresponding to the “flat”, or at least non-systematic, prior in Fig.1c). Moreover, in Experiment 3, half of the faces in the critical Study trials were masked with random, pixel noise, which should reduce the precision of the sensory evidence, and hence reduce PE for these trials (left side of Fig.1c). Orthogonal to this manipulation of perceptual clarity, one half of the faces were presented in a prior Training phase. This “priming” is assumed to increase the precision of the evidence during the critical Study trials, and hence increase PE (right side of Fig.1c). We therefore hypothesized better 3AFC associative memory for the critical Study trials for (1) clear relative to degraded faces and (2) primed relative to unprimed faces. We had no specific prediction about whether these factors would interact (this would depend on whether the changes in precision of the evidence were super-additive or sub-additive).

### Methods

#### Participants

Nineteen participants were recruited using the same procedure as described in Experiment 1. Three participants were excluded because their test performance was at chance. Final analysis included a fully counterbalanced dataset of 16 participants (5 female, mean age 27).

#### Material & procedure

Primed and degraded faces were fully crossed into 4 Study conditions: Primed & Clear (PC), Primed & Degraded (PD), Novel & Clear (NC), Novel & Degraded (ND). Stimuli consisted of 192 colour photographs of indoor and outdoor scenes and 192 black and white photographs of male and female faces. Faces were degraded by replacing a random set (57%) of the image pixels with grey pixels, using MATLAB. Pilot data confirmed that degraded faces were harder to identify, but could still be recognized as the same person as in the clear version. Scene and face stimuli were divided into 4 sets of 48 images and randomly allocated to the 4 conditions (PC, PD, NC, ND), counterbalanced across participants (see Appendix C for more details).

**Fig. 6:**
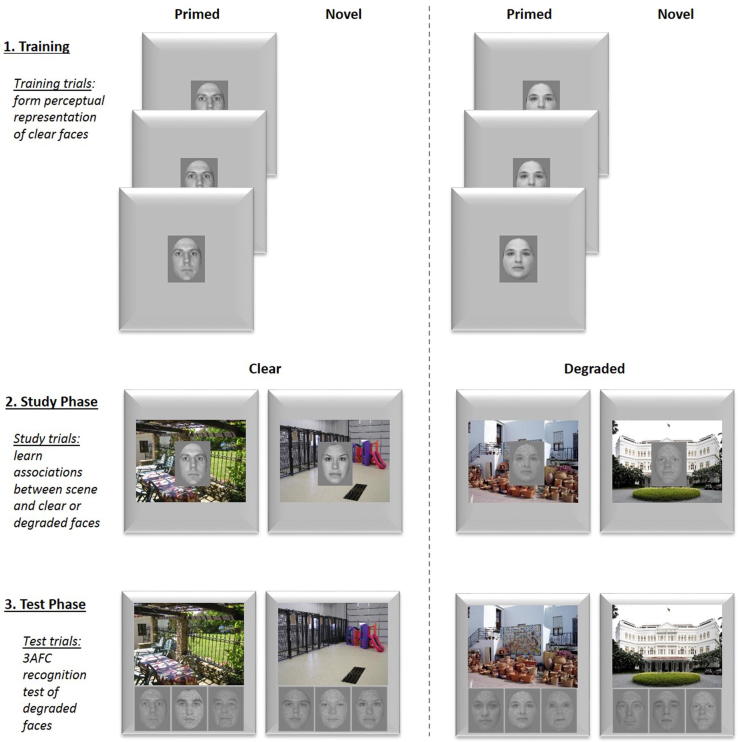
Design of Experiment 3. 12 scene-face pairs were shown per block, in 16 blocks. In both Training and Study phases, the primary task was to judge pleasantness of the faces. In the Training phase all faces were presented clearly. At Study participants were presented with degraded faces of which half were primed (DP) and the other half were not primed/novel (DN) and clear (not degraded) faces, of which half were primed (CP) and the other half was not primed/novel (CN). During Study an occasional target task was to spot scenes containing the moon (not shown). The distractor task was a 15 s odd/even number classification task. Test was 3AFC, self-paced and faces were presented degraded in all conditions.

The experiment was divided into 16 runs, 8 in the clear face condition and 8 in the degraded face condition (face degradation conditions were not mixed within run). Each run contained 6 primed and 6 unprimed faces, each with a unique scene. The procedure for one run is illustrated in Fig. 6 and described below. Participants performed an additional practice block of 5 trials with extra stimuli in order to become familiar with the procedure.

#### Training phase

The Training Phase was introduced to vary the perceptual representation for half of the faces presented at Study via pre-exposing (priming) them. For each run, 6 out of 12 faces were presented three times in pseudorandomised order, with all faces being presented once before being presented again. All faces were presented clearly in this phase. Trials started with a centrally presented fixation cross (500 ms), before a face appeared in the middle of the screen for 1750 ms. In that time period, participants made subjective and speeded judgements whether this face was ‘more pleasant’ or ‘less pleasant’ than average.

#### Study phase

Two different sets of Study runs were created, one presenting scenes paired with degraded faces, of which half were trained/primed (DP) and the other half was not trained/not primed and therefore novel (DN). The other set presented scenes with clear (not degraded) faces, of which half were primed (CP) and the other half was not primed/novel (CN). The presentation of the two sets alternated within participants, and the start order was counterbalanced across participants. The presentation of primed and novel faces within a run was intermixed such that neither primed or novel trials were presented for more than 3 successive trials. Each Study trial started with a scene shown in the centre of the screen for 1000 ms. A face was then superimposed on the scene for another 400 ms followed by a response screen showing a fixation cross for 800 ms. Participants made the same pleasant/unpleasant judgement as in the Training Phase. In order to ensure participants would attend to the scene, they were instructed to press a key if they saw a picture of the moon, which would occur 1–2 times per block. In moon trials, no face appeared, and the image disappeared after 1400 ms, with an 800 ms gap preceding the next trial’s onset, so trial timing was maintained. Moon trials were pseudorandomly interspersed so that they were never consecutive.

#### Test phase

After the Study phase, participants performed an odd/even distractor task for 15 s, to reduce any contribution of short-term memory. Memory for face-scene pairs was assessed using a 3AFC test. After a 300 ms blank screen, a scene was presented with three faces displayed below it. All faces, regardless of study condition, were shown as degraded images, with the pixel noise identical to that used for the same face at Study, such that there was a perceptual study-test match for degraded faces. If anything, this match would boost performance for degraded relative to clear faces, making the predicted advantage for clear faces more difficult to obtain. Face choices came from the same condition, target location was counterbalanced across Test trials, and all three options appeared three times during the Test Phase: once as the target, and twice as a foil, with the order of these occurrences randomised.

#### Analysis

3AFC memory performance was assessed with a 2 × 2 ANOVA, in which we predicted main effects of degradation and of priming. Given our specific hypotheses, follow-up pairwise *t*-tests were conducted for the simple effects of degradation under each level of priming, and the simple effects of priming under each level of degradation.

### Results

Performance on the associative memory 3AFC test is shown in Table 1. As expected, performance was best for conditions with higher PE owing to more precise sensory evidence (i.e., clear and primed conditions). The 2 × 2 ANOVA revealed a significant main effect of degradation, *F*(1, 15) = 12.75, *p* = .003, and of priming *F*(1, 15) = 11.42, *p* = .004. The interaction was not significant, *F*(1, 15) < 1, *p* = .561. Pairwise *t*-tests confirmed that associative memory for clear faces was superior to degraded faces, whether primed, *t*(15) = 2.15, *p* = .05, *d* = .48 (mean difference: .06; 95% confidence interval from .0004 to .12), or unprimed, *t*(15) = 3.68, *p* = .002, *d* = .67 (mean difference: .08; 95% confidence interval from .03 to .12). Furthermore, associative memory for primed faces was superior to novel faces, whether presented clearly, *t*(15) = 2.42, *p* = .03, *d* = .48 (mean difference: .06; 95% confidence interval from .01 to .12) or degraded, *t*(15) = 3.05, *p* = .01, *d* = .76 (mean difference: .08; 95% confidence interval from .02 to .14).

### Discussion

According to PIMMS, Experiment 3 manipulated the precision of the sensory evidence for a face, to see if this affects the ability to form a new association between that face and an (unpredictive) scene. As hypothesized, memory for the new scene-face pairing was better when the evidence was more precise, consistent with a larger prediction error. This memory advantage was reflected by significantly better 3AFC associative memory performance for primed over novel faces, as well as clear over degraded faces.

Faces would be expected to be processed more fluently when primed relative to unprimed, and when clear relative to degraded. One might generally expect more fluent perception to lead to better memory. It is important to note, however, that we tested memory for the scene-face association, not for the face itself (and both 3AFC foils were studied in the same manner, i.e., equally fluent). Moreover, while one might expect perceptually degraded images to be worse remembered, the test faces in the 3AFC were also degraded with the same noise used in the study phase, which, if anything, should favour memory for the degraded faces, in terms of study-test perceptual match. Furthermore, these findings did not require manipulating the priors by training different scene-face pairings, and so cannot reflect differences in item familiarity or proactive interference. We consider the PIMMS account, and other potential interpretations such as attentional resources, in the General Discussion, in the context of all three experimental designs used here.

## General discussion

The present series of experiments support our hypothesis that predictions, specifically prediction errors, play a role in one-shot, declarative memory in humans. We systematically manipulated prediction errors by changing: the difference between the mode of the prior and the evidence distribution (Experiment1), the precision of the prior, while keeping the difference between the mode of prior and evidence constant (Experiment 2a–c) and the precision of the evidence, without any prior expectations (Experiment 3).

We found a consistent pattern across experiments, whereby associative memory for a single, arbitrary pairing of a scene with a novel word or face was best when that word/face was least predicted by prior expectations. More specifically, memory for a new scene-word pairing in Experiment 1 was better when the scene was previously associated with a different category of words, relative to when it was associated with the same category of words. Likewise, memory for a new scene-face pairing in Experiments 2a–c was best when the scene was strongly associated with a different face, relative to being only weakly associated with a different face. Finally, in Experiment 3 memory was best for new scene-face pairs when faces were presented more clearly and when they were previously primed.

All of these findings are in keeping with the PIMMS framework (Henson & Gagnepain, 2010), in which prediction error drives all types of memory. Given the long tradition of paired associate learning in humans, and the history of PE in animal learning (Pearce and Hall, 1980, Rescorla and Wagner, 1972, Schultz et al., 1997), it may seem surprising that findings like ours have not been reported before (to our knowledge). We suspect that one reason for this relates to our use of 3AFC to measure associative memory; specifically our use of response options that did not include items from the Training phase, but only used items from the critical Study trials. This is important in order to control for “proactive interference” of associations from the Training phase (Keppel and Underwood, 1962, Postman, 1962, Underwood, 1957). For example, had we included Trained items among the response options, or used cued recall instead (as typical of many human paired associate studies), then we expect that memory performance in Experiment 1 would actually be worse (rather than better) in the High PE than Low PE conditions, because participants would use their knowledge about the valence associated with each scene to bias their choices towards words congruent with that valence (a “congruency” advantage; Craik and Tulving, 1975, Hall and Geis, 1980, Schulman, 1974). Likewise, we expect that memory performance in Experiment 2a–c would actually be worse in the High PE condition than the Baseline condition, because in the High PE Training trials, the face was paired multiple times with the same scene, and so would be more likely to win any response competition during the test phase against a face paired only once.

Nonetheless, there are alternative explanations for the results in each of our experiments, which we consider below.

### Feedback-driven learning

An alternative explanation for the results of Experiment 1 is that participants learned better when they received explicit, negative feedback (i.e., an explicit error signal), which occurred on most critical trials in the High PE condition, but on very few trials in the Low PE condition. This feedback-driven account, however, would seem unable to explain the results in Experiment 2a–c and 3, where there was no explicit feedback given; the participant’s task was only to make an easy, binary and/or subjective decision about the face.

### Stimulus associability

A possible alternative interpretation of the findings in Experiments 2a–c is that participants abandoned attending to scenes that were not predictive (in the Low PE condition), and/or paid extra attention to scenes that were highly predictive (in the High PE condition). This relates to the idea of stimulus “associability” (Beesley and Le Pelley, 2011, Le Pelley, 2004, Le Pelley and McLaren, 2003). This idea, originally introduced by Mackintosh (1975), proposes that in addition to associating a stimulus with a specific reward, animals update the associability of that stimulus as a function of its past history of pairings with rewards (i.e., associability is a property of the stimulus itself, rather than stimulus-reward association). It is possible that the associability of scenes in the High PE conditions of Experiments 2a–c became higher than those in the Low PE or Baseline conditions, explaining the stronger association formed when those High PE stimuli were paired with a new face. However, such an associability account could not explain the results of Experiment 1 or Experiment 3. In Experiment 1, the scenes in the High PE and Low PE conditions had identical associative histories up to the critical Study trial, so could not differ in associability at the point of encoding. Equally, Experiment 3 did not pair any stimuli prior to the critical Study phase, so that differences in associability could not emerge.

### Other associative interference accounts

As previously mentioned in the Discussion of Experiment 2a, the difference between High and Low PE conditions in that experiment could reflect interference during Study and/or during 3AFC test, from prior associations established in the Training phase: specifically, a greater number of competing associations might occur in the Low PE condition. Although our 3AFC test controls for overt proactive interference, in the sense that prior associates from the Training phase are not response options, those associates may still come to mind covertly, and interfere with ongoing processing. A similar type of interference might have occurred during the test phase of Experiment 1, where test trials from the Consistent condition would have one more competing word that comes to mind by virtue of being consistent with the trained words. However, this interference account would seem unable to explain the memory advantage for primed and clear faces in Experiment 3 (where there was no prior association), and more importantly, would actually predict the opposite pattern of results when comparing the High PE and Baseline conditions of Experiment 2b–c, i.e., worse, not better, performance in the High PE condition, because the High PE condition had more repeated pairings than the Baseline conditions.

### Resource-based/attentional accounts

An alternative explanation of the results of Experiment 3 is that primed faces, and faces without noise, require fewer “processing resources”. This then frees up resources from processing the face itself, allowing those resources to better encode the face-scene association instead. This resembles the “Item-Context” trade-off account of dissociations between item and source memory that was proposed by Jurica and Shimamura (1999). The same type of explanation could apply to the results of Experiments 1, 2a–c, if it is assumed that items that are predicted by a cue (scene) require fewer resources.

A related idea is that unpredicted stimuli engage greater selective attention, and it is this increase in attention that drives memory encoding. However, both the resource and attentional accounts appear to be insufficient explanations, because there must be an initial cause of the change in attention/resources in Experiments 1, 2a–c. It seems likely that this cause arises when a prediction is not fulfilled, i.e., by appealing to the concept of PE. It is possible that attention/resources *mediate* the relationship between PE and one-shot associative encoding – e.g., that without the increased attention triggered by PE, that PE does not translate into improved memory. This might be tested by future experiments that factorially manipulate the amount of attentional resources (e.g., in dual-task paradigms), or with other methods like electroencephalography that can temporally dissociate PE from subsequent attentional changes.

## Conclusion

In summary, although there may be possible alternative accounts for the individual results of Experiment 1, Experiments 2a–c and Experiment 3, like those outlined above, the prediction error account of the PIMMS framework would seem the most parsimonious account when taking all findings together. Indeed, the experimental designs, and the predictions for their outcomes, were generated *a priori* by the PIMMS framework. While future work may show that prediction error on its own is not sufficient, in that increased attention that follows a prediction error may be needed to encode stronger memories, the concept of PE appears central to explain the present findings.

While the present concepts of unimodal distributions for a single prior and single evidence distribution in Fig. 1 are likely too simplistic, the PIMMS framework is an example of recent Bayesian approaches that offer a more general, unifying framework for explaining many aspects of human and animal learning (Courville et al., 2006, Dayan et al., 2000, Gallistel and Gibbon, 2000, Gershman and Niv, 2012). Unlike purely associative theories, Bayesian models assume that animals acquire a ‘world model’, reflecting statistical regularities between stimuli, outcomes and contexts (Barto, Mirolli, & Baldassarre, 2013). For example, if stimuli are highly predictive of outcomes in one context, but not in another context, then the learning of the same pairings should differ across contexts, with less learning occurring in the less predictive context, corresponding to “expected uncertainty” (Yu & Dayan, 2005). According to PIMMS, contexts, stimuli and outcomes (and potentially further levels of representation) correspond to hierarchical levels within a neural perceptual processing pathway (Henson & Gagnepain, 2010). The concept of priors, evidence and prediction errors (at each level of this hierarchy), as presented by the PIMMS framework, therefore, may offer a general way of understanding these empirical phenomena and their neural bases.

## Figures and Tables

**Table 1:** Mean (standard error in brackets) of performance in the associative 3AFC test (chance = .33) across all Experiments (high PE performance in Experiment 2b–c is collapsed across 4 and 6 repetition conditions; see text).

Experiment	High PE	Baseline	Low PE
Experiment 1	.66 (.031)	–	.60 (.023)

Experiment 2a	.50 (.026)	.49 (.022)	.45 (.024)
Experiment 2b	.67 (.033)	.64 (.035)	–
Experiment 2c	.58 (.024)	.55 (.023)	–

Experiment 3	Primed		Unprimed
Clear	.74 (.035)	–	.67 (.030)
Degraded	.68 (.025)	–	.60 (.028)

## Appendix A Supplementary material

Supplementary Appendices A–C
