# Supplementary material for: Does prediction error drive one-shot declarative learning?
Source: J Mem Lang. 2017 Jun;94:149–65. doi: 10.1016/j.jml.2016.11.001 (PMC5381756; doi:10.1016/j.jml.2016.11.001)
Supplement: Supplementary Appendices A–C [file mmc1.docx]

# Appendices

Appendix A

In Set 1 scenes were randomly paired with one of two words: half were paired with the word ‘positive’ and the other half with the word ‘negative’. In Set 2-5 scenes were paired with one of eight words: four words with positive valence (‘calm’, ‘happy’, ‘lucky’, ‘relaxed’) and four words with negative valence (‘panic’, ‘sad’, ‘stressed’, ‘anxious’). The eight words repeated within and across Sets, with the constraint that across Sets distinct images of the same category were never paired with the same word. Importantly, the valence of words in word-scene pairs was the same for images of the same category in Sets 1-5 and hence the scene category became a reliable predictor for the valence of the word it was paired with. Finally, Set 6 paired scenes with 48 unique words of positive or negative valence. Half of the pairings were consistent in valence with Set 1-5, while the other half was inconsistent (i.e., scenes were now paired with a word of the opposite valence). The Sets were allocated to different phases of learning: Familiarisation (Set 1), Training (Set 2-3), Study (Set 4-5) and critical Study (Set 6).

Appendix B

To ensure counterbalancing of Training and Study trials across conditions, the scenes were divided into 3 sets of 36 photos, while the faces were split into 3 groups: one for Training trials (T) common to all conditions, itself containing 3 sets of 36 photos; one for critical Study trials (S) common to all conditions, itself containing 3 sets of 36 photos; and one for the additional Training trials (aT) needed for the Low PE condition, containing 5 sets of 36 photos. Group ‘T’ faces were randomly paired with the 3 sets of scenes, to form 3x36 scene-face Training trials, with the assignment of the 3 sets of 36 scene-face pairs to the three conditions counterbalanced across participants. Group ‘S’ faces were also randomly paired with the same 3 sets of scenes, to form 3x36 new scene-face Study trials. The assignment of Study faces to condition was rotated independently of the assignment of Training faces to condition. The 5x36 faces of Group ‘aT’ were used as the first to fifth associate paired during Training with the 36 scenes in the Low PE condition (followed by the sixth associated face created by the Group ‘T’ Training pairs, described above).

Appendix C

The scenes were divided into 2 Sets of 48 photos, while faces were split into 4 Sets of 48 photos. During training the 2 Sets of scenes were randomly paired with 2 Sets of faces to form 2x48 scene-face pairs (one Set per condition). At critical Study the 2 Sets of scenes presented during training were randomly paired with the remaining 2 Sets of faces not shown at training. The assignment of Study faces to condition was counterbalanced and rotated independently of the assignment of Training faces to condition.
